# ER remodeling via ER-phagy

**DOI:** 10.1016/j.molcel.2022.02.018

**Published:** 2022-04-21

**Authors:** Andrea Gubas, Ivan Dikic

**Affiliations:** 1 Institute of Biochemistry II, Faculty of Medicine, Goethe University Frankfurt, Frankfurt, Germany; 2 Buchmann Institute for Molecular Life Sciences, Goethe University Frankfurt, Frankfurt, Germany; 3 Max Planck Institute of Biophysics, Frankfurt, Germany

## Abstract

The endoplasmic reticulum (ER) is a hotspot for many essential cellular functions. The ER membrane is highly dynamic, which affects many cellular processes that take place within the ER. One such process is ER-phagy, a selective degradation of ER fragments (including membranes and luminal content), which serves to preserve the size of ER while adapting its morphology under basal and stress conditions. In order to be degraded, the ER undergoes selective fragmentation facilitated by specialized ER-shaping proteins that also act as ER-phagy receptors. Their ability to sense and induce membrane curvature, as well as to bridge the ER with autophagy machinery, allows for a successful ER fragmentation and delivery of these fragments to the lysosome for degradation and recycling. In this review, we provide insights into ER-phagy from the perspective of membrane remodeling. We highlight the importance of ER membrane dynamics during ER-phagy and emphasize how its dysregulation reflects on human physiology and pathology.

## Graphical abstract

**Figure:**
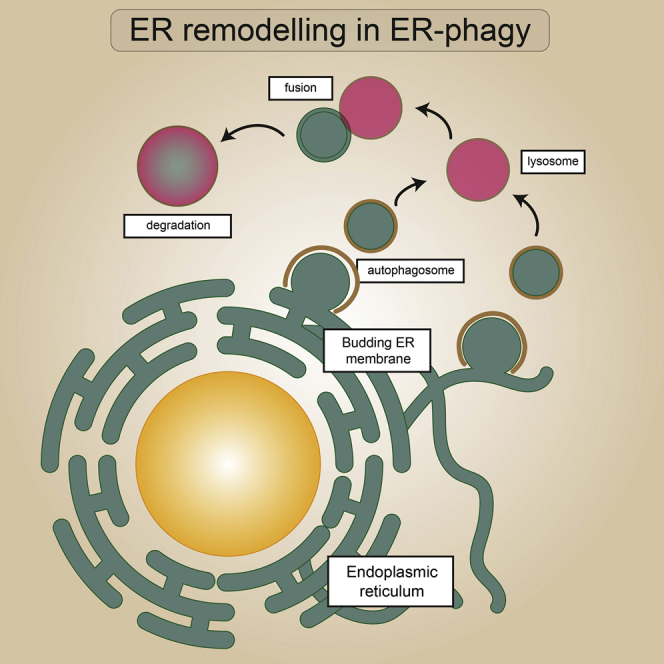

## Teaser

In this review, Gubas and Dikic highlight the importance of ER membrane remodeling during the process of selective ER degradation. The authors focus on reticulon-homology-domain-containing proteins and their involvement in ER membrane budding and vesicle formation.

## Introduction

The endoplasmic reticulum (ER) is the most extensive continuous endomembrane system in cells. It emerges from the nuclear membrane, expands throughout the cell, and forms connections with other cellular organelles. The ER has important functions in protein and lipid synthesis, their transport, ion homeostasis, and inter-organelle communication. The morphology of the ER is complex and highly dynamic, and subject to constant remodeling. In order to facilitate these distinct functions, the ER has developed different structural compartments, including the nuclear envelope, ER sheets, and ER tubules. ER sheets are predominantly studded with ribosomes and are, therefore, also known as the rough ER. Smooth, highly dynamic ER tubules are connected by three-way junctions and are mainly localized at the cell periphery (Borgese et al., 2006; De Brito and Scorrano, 2008; Shibata et al., 2006; Walter and Ron, 2011).

**Figure 1:**
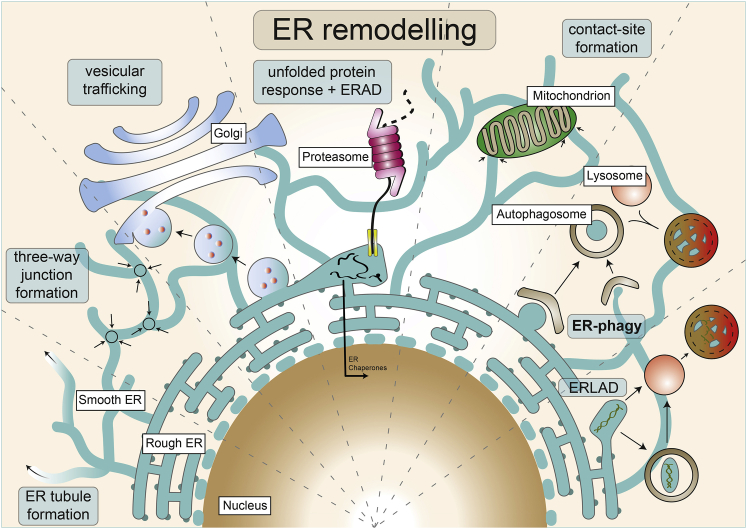
Schematic representation of processes relying on ER remodeling Some of the processes that rely on ER remodeling as part of their mechanism (highlighted in light blue), separated by the dashed lines, even though in reality they do overlap in most cases. From left to right, ER tubule formation, three-way junction formation, vesicular trafficking, unfolded protein response and ERAD, ER-organelle contact-site formation, ER-phagy and ERLAD are only some of the processes essential for cellular function, which require or induce changes to ER morphology.

ER remodeling (or ER re-shaping) is defined as dynamic changes to ER morphology and has been observed across different cell lines, under different stimuli, as well as during normal conditions of cellular homeostasis (Pendin et al., 2011a). ER remodeling is central to ER membrane/vesicle formation and trafficking, as well as adaptation to stress and after-stress recovery (Figure 1). This provides the cell a method of ER quality control and homeostasis maintenance. Processes such as vesicle formation, collagen degradation, and the unfolded protein response (UPR) are reliant on such quality control. One of these mechanisms of quality control is the selective autophagy of the ER, termed ER-phagy (from the Greek phagein, meaning “to eat”) (Dikic, 2018; Wilkinson, 2019).

Briefly, autophagy is a catabolic process involved in maintaining cellular homeostasis. Autophagy is a process of lysosomal degradation, in which excess, damaged, or misfolded proteins, damaged organelles, pathogens, and aggregates are degraded into nutrients that cells can re-use as building blocks. Autophagy can be non-selective, where bulk portions of cytoplasm are sequestered into the vesicle formed *de novo*, termed the autophagosome, for their subsequent lysosomal degradation to replenish cellular nutrients. Non-selective autophagy is frequently induced upon stress, when nutrients are low. To maintain the number and integrity of cellular organelles or to rid the cell of misfolded proteins, aggregates, and pathogens upon an infection, the cell employs selective autophagy. Selective autophagy cargo can include fragments of the ER (ER-phagy), mitochondria (mitophagy), lysosomes (lysophagy), lipid droplets (lipophagy), protein aggregates (aggrephagy), bacteria and viruses (xenophagy), and many more (reviewed in the studies conducted by Johansen and Lamark, 2020; Kirkin and Rogov, 2019; and Stolz et al., 2014).

ER-phagy has been recognized as a major pathway relying on ER remodeling for its function. It is defined as a form of selective autophagy that enables lysosomal degradation of distinct ER components. ER-phagy is active in basal, non-stress conditions, thereby maintaining the size of the ER (Chino and Mizushima, 2020; Dikic, 2018; Grumati et al., 2018; Molinari, 2021). ER-phagy is up-regulated upon stress, such as perturbations in calcium levels, accumulation of misfolded proteins, or changes in redox potential. As part of the recovery process, ER-phagy can degrade protein aggregates within the ER-lumen as well as trim excess ER membranes generated as a response to stress.

**Figure 2:**
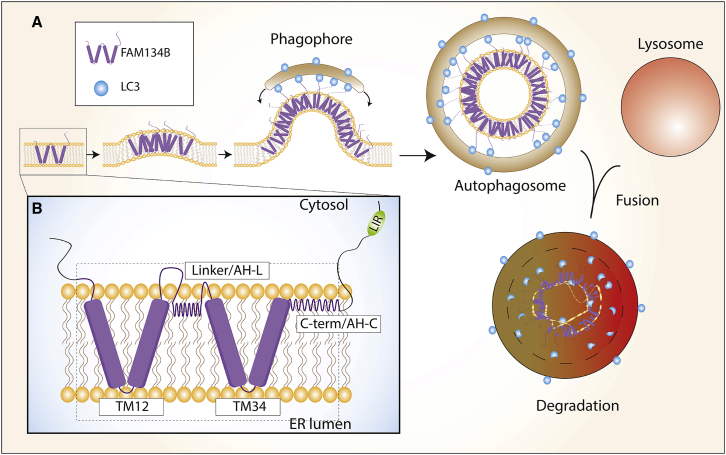
Schematic representation of ER-phagy and reticulon-homology domains (A) FAM134B, the best characterized RHD-containing ER-phagy receptor, can oligomerize, sense, and induce membrane curvature. High concentration of FAM134B leads to membrane budding, which is recognized by the autophagy machinery through direct binding between FAM134B and LC3/GABARAP proteins. This binding provides additional forces that lead to scission of these ER membrane buds and their subsequent incorporation into the forming autophagosome. The autophagosome will fuse with a lysosome, where the fragmented ER membrane and embedded RHD-containing proteins are degraded. (B) RHDs assume a wedge-shaped form when inserted into the ER membrane. RHDs consist of two conserved transmembrane hairpins (TM1,2 and TM3,4) and two amphipathic helices (AH-L and AH-C). AH-L is part of a linker sequence, connecting TM1,2 and TM3,4, and AH-C is localized C-terminally of TM3,4. The transmembrane hairpins locally compress the lipid bilayer, while the amphipathic helices allow stretching of the cytosolic leaflet to induce strong local membrane deformations.

ER-phagy receptors directly link the ER cargo and autophagic machinery, resulting in their subsequent degradation along with the cargo (Gubas and Dikic, 2022). ER-phagy receptors contain at least one LC3-interacting region (LIR) that enables a direct interaction with autophagy modifiers LC3 (microtubule-associated protein 1A/1B-light-chain 3) and GABARAP (GABA Type A receptor-associated protein) (Chino and Mizushima, 2020; Dikic, 2018; Grumati et al., 2018; Molinari, 2021). Interestingly, some of the ER-phagy receptors were initially identified as ER-membrane-shaping proteins, highlighting the importance of ER remodeling in functional ER-phagy (Figure 2A).

In this review, we turn our attention to the remodeling events in ER-phagy, summarizing the relevant and sparse knowledge accumulated in the past years. We will emphasize the importance of ER remodeling during ER-phagy processes, describe the proposed mechanism of vesicle formation, and highlight the contribution of individual ER-phagy receptors toward membrane re-shaping.

### ER dynamics and remodeling

To meet cellular needs and adapt to stress conditions, the ER dynamically changes its morphology. A process as common as cell shape change or cell migration would require major changes to ER morphology—including rearrangement, budding, scission, and fusion of ER membranes (Figure 1). The formation of the tubular ER network is facilitated by evolutionary conserved membrane-shaping protein families reticulons (RTNs) and receptor expression enhancing proteins (REEPs), which are enriched on highly curved ER membranes—ER tubules and the edges of ER sheets (Voeltz et al., 2006). Removal of these proteins results in increased sheet formation, but overexpression of at least one of them can restore the tubular morphology (Hu et al., 2008; Shibata et al., 2009; Voeltz et al., 2006; Wang et al., 2021). Furthermore, atlastins (ATLs) mediate the fusion of ER tubules, resulting in the formation of tubular interconnections, which are also known as three-way junctions (reviewed in the study conducted by Wang and Rapoport, 2019).

ER stress is sensed by pathways such as the UPR, which induces chaperone production to facilitate correct protein folding. When unsuccessful, misfolded proteins are generally exported from the ER into the cytosol, where they undergo degradation within the proteasome, a process called ER-associated degradation (ERAD). The UPR simultaneously upregulates lipid production, which contributes toward the stress-induced expansion of the ER. Providing sufficient space to prevent protein overcrowding has been shown to alleviate the stress (Schuck et al., 2009). Along with promoting chaperone and lipid biogenesis, the UPR also signals for the production of some autophagy-related genes, thereby inducing ER-phagy (Bernales et al., 2007) (for more details on UPR, please refer to Wiseman et al., [2022] in this issue of *Molecular Cell*). ER-phagy is hypothesized to fulfill different functions in the course of ER stress—initially, it might facilitate degradation of luminal protein aggregates, and later, it might aid the ER in shrinking back to its original size by degrading excess membranes.

Dynamic changes to ER morphology allow for flexible physical and functional connections to other cellular organelles. These connections are considered to be central hubs for coordinating cellular physiology. Some of the well-characterized contact sites include ER-mitochondria, which facilitate direct transfer of lipids and Ca^2+^ and play important roles in cholesterol metabolism and phospholipid homeostasis; ER-plasma membranes, which regulate Ca^2+^ levels and phosphatidylinositol metabolism; and ER-Golgi, which enable the transfer of secreted lipids and proteins (reviewed in the study conducted by English and Voeltz, 2013).

Cells with defects in ER shaping and remodeling processes are unable to fulfill the essential ER functions, fail to respond to changing cellular needs, and cannot resolve ER stress. ER stress has been linked to the development of numerous human diseases, including infections, neurodegeneration, and cancer (Bergmann et al., 2017; Cabral-Miranda and Hetz, 2018; Choi and Song, 2019). In order to prevent disease onset and maintain its integrity, the ER has developed a number of quality control and stress-coping mechanisms, with the re-shaping and remodeling of the ER membrane taking center stage.

### The role of microtubules in ER remodeling

An early idea behind the mechanisms of ER dynamics involved microtubule and actin filament rearrangements, given the close association of ER tubules and microtubules (reviewed in the study conducted by Du et al., 2004). And while the cytoskeleton does provide a certain degree of dynamics during ER tubule formation and membrane rearrangement, it is not indispensable. The formation of ER tubules, membrane rearrangement, or branching of the tubules generally follows the path of microtubules via two separate mechanisms—the sliding mechanism, where the ER tubule associates with motor proteins and slides along the microtubules, and the tip attachment complex (TAC) mechanism, where ER tubules are attached to the tip of the polymerizing microtubules (Waterman-Storer and Salmon, 1998). Finally, the pioneering work by Tom Rapoport’s group highlighted the importance of reticulon and reticulon-like proteins in the process of ER dynamics (Voeltz et al., 2006).

Nevertheless, the cytoskeleton continues to be linked with the topic of ER dynamics. More recently, another mechanism of ER tubule formation was discovered, termed hitchhiking. Hitchhiking describes a process where ER tubules are formed by tethering to highly motile organelles such as endosomes or lysosomes, which themselves use microtubules and protein motors for their transport. While the underlying mechanism of this process remains unknown, it is considered to be transient or reversible, allowing the forming tubules to fuse with other ER tubules, or to withdraw in case of tether breakage during ER tubule extension (Guo et al., 2018).

How the cytoskeleton contributes to ER-phagy is one of the open questions in the field. In yeast, the actin cytoskeleton has been found to be required for localization of Atg40-containing ER fragments within the cell (Chen et al., 2018). However, the principal mechanisms and the regulation of this process are currently lacking.

## ER-phagy

Autophagy is up-regulated during stress, such as nutrient starvation, when it tries to secure enough nutrients for the cell to cope during the stress conditions and is under the control of the target of rapamycin (TOR) kinase, its key regulator. TOR inhibition results in the activation of a cascade of autophagy kinase complexes, including Atg1/ULK1 complexes and the class III Vps34 lipid kinase complex, leading to the production of phosphatidylinositol (III) phosphate (PI3P) at the ER exit sites, initiating *de novo* formation of a double membrane phagophore. During the phagophore expansion, Atg8/LC3/GABARAP proteins undergo lipidation onto the phosphatidylethanolamine (PE) within the forming phagophore, simultaneously surrounding the cargo marked for degradation. This step is facilitated by autophagy receptors. Phagophore closure results in the formation of an autophagosome, which will fuse with a lysosome in order for the cargo to be degraded and recycled back into the cytosol (reviewed in the studies conducted by Farré and Subramani, 2016 and Mercer et al., 2018).

The term ER-phagy was first coined in 2006 (Bernales et al., 2006) to describe selective degradation of the ER through autophagy, but we started gaining a deeper understanding into the process with the discovery of ER-phagy receptors—mammalian FAM134A, B, and C (Khaminets et al., 2015; Kumar et al., 2021; Reggio et al., 2021), Sec62 (Fumagalli et al., 2016), RTN3 (Grumati et al., 2017), CCPG1 (Smith et al., 2018), ATL3 (Chen et al., 2019), TEX264 (An et al., 2019; Chino et al., 2019), CALCOCO1 (Nthiga et al., 2020), and C53 (Stephani et al., 2020); yeast Atg39, Atg40 (Mochida et al., 2015), and Epr1 (Zhao et al., 2020); and plant ATI1, ATI2, and ATI3 (Honig et al., 2012; Michaeli et al., 2014; Zhou et al., 2018), Rtn1 and Rtn2 (Zhang et al., 2020), Sec62 (Hu et al., 2020), and C53 (Stephani et al., 2020). All hitherto identified ER-phagy receptors contain an ATG8-interacting motif (AIM), or LIR in mammals, and are localized to the ER. Generally, we can split ER-phagy receptors into two groups—(1) reticulon homology domain (RHD)-containing receptors (such as mammalian FAM134 proteins and RTN3, yeast Atg40, or plant Rtn proteins) and (2) receptors not containing RHD (such as mammalian Sec62, TEX264, or C53; yeast Atg39; or plant C53). Receptors corresponding to the first group are often involved in ER fragmentation and membrane remodeling, which was not observed with the others to the same extent.

ER-phagy can also be micro-ER-phagy, where ER components are directly engulfed by the lysosome without the assistance of autophagosomes. This type of ER-phagy is frequently associated with recovER-phagy, where the translocon component Sec62 takes on the role of the cargo receptor and, under the control of ESCRT-III complex machinery, drives the engulfment by the lysosomes (Loi et al., 2019).

### Membrane-shaping ER-phagy receptors

Portions of the ER that are targeted for degradation are recognized by the autophagy machinery through ER-phagy receptors. These receptors generally undergo oligomerization in order to facilitate targeting of the bulky cargo (Gubas and Dikic, 2022). ER-phagy receptors, with the exception of CALCOCO1 and C53, contain transmembrane domains, anchoring them to the ER membrane. CALCOCO1 and C53 are indirectly recruited to the ER—CALCOCO1 binds ER-resident VAP proteins (Nthiga et al., 2020), whereas C53 forms a ternary complex with UFMylation E3 ligase UFL1 and its ER-localized adaptor DDRGK1 (Stephani et al., 2020).

Currently, there are eight mammalian ER-phagy receptors, with that number likely to rise in the near future (for more detailed insight into their function, please refer to the studies conducted by Chino and Mizushima (2020), Molinari (2021), and Wilkinson (2020). While the exact reason for this is unknown, it is likely linked to the variable morphology of the ER in order to facilitate the degradation of different ER subdomains. Likewise, ER-phagy receptors are also differently expressed in specific mammalian and plant tissues. Moreover, ER-phagy receptors have additional roles in cellular quality control mechanism beyond bridging the cargo with the autophagy machinery. It is also likely that different receptors will respond to different types of stress stimuli. With this in mind, only some of these receptors have the capacity to induce significant ER remodeling (such as mammalian FAM134 family, RTN3L and ATL3, and yeast Atg40) (Chen et al., 2019; Grumati et al., 2017; Khaminets et al., 2015; Mochida et al., 2015). One of the main features of these proteins is a hairpin-like domain anchored into the lipid bilayer. In FAM134A-C and RTN3L, two hairpin-like domains are connected via a linker, forming a so-called RHD (Figure 2B). The linker forms an amphipathic helix (AH) in connection with the lipid bilayer. Carboxy-terminally to the second transmembrane domain, the RHDs contain a second AH. Both of these amphipathic helices assist in the function of RHDs (Bhaskara et al., 2019). RHDs serve to retain these proteins within the ER membrane, but their main function is to promote membrane curvature and drive the ER membrane remodeling (Voeltz et al., 2006). This is particularly important for ER-phagy, where the RHD contributes toward the budding and scission of ER membranes for their subsequent degradation. Interactions between the ER bud-localized RHDs and the phagophore provide an additional force in the bud scission, allowing the sequestration of the ER bud within the autophagosome (Figure 2A).

Yeasts have been shown to require another ER-shaping protein for functional ER-phagy—Lunapark 1 (Lnp1), from a family of Lunapark proteins (Chen et al., 2018). Lnp1 is required for successful distribution of Atg40 in ER-derived autophagic puncta upon ER-phagy induction. While the mechanism underpinning Lnp1’s function in ER-phagy remains unclear, the data point toward destabilization of the interaction between Atg40 and its scaffold protein Atg11 upon the depletion of Lnp1, an event required for functional ER-phagy (Chen et al., 2018).

### RHDs in ER-phagy and ER remodeling

FAM134B is the most studied protein in terms of its dual role in ER remodeling and ER-phagy. The FAM134B-RHD, with the assistance of amphipathic helices, can sense areas of high membrane curvature (such as the edges of ER sheets) and form clusters in those areas (Bhaskara et al., 2019). FAM134B oligomerizes through its RHD domain, thus contributing to membrane deformation. Such clustering leads to the formation of FAM134B-positive buds, decorated with LC3 through their direct interaction with the FAM134B LIR, forming a bridge between the ER cargo and the phagophore. Interactions between FAM134B and LC3 provide an additional force for ER bud scission, which will result in ER membrane fragmentation and eventually their delivery to the lysosome for degradation (Figure 2A; Bhaskara et al., 2019; Siggel et al., 2021). ATL2 could also have a role in facilitating the scission of the buds. ATL2 binds FAM134B and likely contributes toward the formation of FAM134B-positive clusters; however, it plays a role downstream of FAM134B, as observed by its depletion abrogating FAM134B overexpression-induced ER-phagy (Liang et al., 2018). Oligomerization of selective autophagy receptors has frequently been observed to be driven by additional factors such as GTP load or post-translation modifications (Gubas and Dikic, 2022). Upon ER stress, phosphorylation of FAM134B by the calcium/calmodulin-dependent protein kinase type II beta (CAMK2B) at S151 drives its oligomerization, thereby positively regulating ER fragmentation and ER-phagy (Jiang et al., 2020). FAM134B is also transcriptionally regulated through TFEB and TFE3 transcription factors, inducing its expression and thereby promoting membrane remodeling and ER-phagy (Cinque et al., 2020). Furthermore, ATLs utilize their GTPase activity and dimerize in GTP-bound form. This process underpins the subsequent fusion of membranes, which is particularly important for branching of the tubules, by forming three-way junctions, one of the main functions of ATLs in ER remodeling (Moss et al., 2011; Orso et al., 2009; Pendin et al., 2011b). ATLs have also been shown to remodel ER membranes in order to facilitate the delivery of FAM134B-positive ER fragments into the autophagosomes, resulting in their own degradation within the lysosome (Liang et al., 2018).

Recently, FAM134A and FAM134C have both been shown to induce membrane budding, albeit at slower rates than FAM134B. Subsequently, both FAM134A and C can drive ER fragmentation in a LIR-dependent manner, promoting ER-phagy (Reggio et al., 2021).

In yeast, Atg40, which is closely related to FAM134B, undergoes homodimerization, as well as sensing and inducing membrane curvature through its RHD. Atg40 homodimers assemble on the ER membrane, creating highly curved membrane buds, which are eventually surrounded by the phagophore. The phagophore is stabilized around the bud through direct interaction between Atg40 and Atg8, trapping the Atg40 homodimers. As the phagophore elongates, the ER membrane is further bent through the Atg40 super-assembly, allowing efficient packaging of ER membrane within the newly formed autophagosome (Mochida et al., 2020). These ER fragments are subsequently delivered to the lysosome and degraded. In plants, maize Rtn1 and Rtn2 have been shown to regulate ER fragmentation in a dose-dependent manner, likely pointing toward a similar mechanism to mammalian FAM134 proteins (Zhang et al., 2020).

Another ER-phagy receptor, RTN3L, predominantly resides on highly curved tubules. We previously reported that RTN3L homo-oligomerization results in fragmentation of the tubular ER. These fragments are positive for LC3 and are eventually delivered to and degraded in the lysosome. RTN3L contains six LIRs, which have not been identified in other RTN family proteins. Interestingly, RTN3L is the only RTN protein that can induce fragmentation of ER tubules upon amino acid starvation, and mainly in its homo-oligomerized form. Hetero-oligomerization between RTN3L and its shorter isoform RTN3S leads to enhanced tubulation of the ER (Grumati et al., 2017). RTN3L and FAM134B have also been found to interact with other RHD-containing proteins (Grumati et al., 2017), likely pointing toward formation of large super-molecular clusters, which would provide a strong base for membrane remodeling in ER-phagy.

RTN3L has recently been found to be recruited to ER-endosome membrane contact sites through Rab9A, tethering endosomes to the ER and thereby regulating endosomal maturation and cargo sorting (Wu and Voeltz, 2021).

## ER-to-lysosome-associated degradation (ERLAD)

ER-to-lysosome-associated degradation (ERLAD) is an overarching term defining the degradation of proteasome-resistant misfolded proteins and aggregates. One of the main reasons why some proteins escape proteasomal degradation is their size; for example, pancreatic enzyme aggregates (Smith et al., 2018), collagen (Forrester et al., 2019), α1-antitrypsin (Fregno et al., 2018), and others (Parashar et al., 2021; Fregno and Molinari, 2019; Figure 1). Interestingly, distinct ERLAD cargos will be delivered to the lysosome in a way that is specific to that cargo, which will not always be via macroautophagy but might also be microautophagy or vesicular transport. Collagen, a major component of bone and cartilage (Bateman et al., 2009), is synthesized in the ER and folded into triple helices before being exported out of the ER with the assistance of COP-II proteins and TANGO1 (Malhotra and Erlmann, 2015; Raote et al., 2018). A failure in folding or secretion results in collagen degradation within the lysosome (Ishida et al., 2009). Interestingly, FAM134B binds to misfolded procollagen through the interaction with Calnexin (CANX), a transmembrane ER chaperone, which in this case represents a co-receptor. Recruitment of FAM134B facilitates LC3-dependent autophagic degradation of misfolded procollagen (Forrester et al., 2019). The mechanism of membrane remodeling in this process is yet to be determined; however, FAM134B likely drives membrane vesiculation to facilitate the delivery of procollagen into the autophagosome. This is supported by correlative light-electron microscopy (CLEM) analysis, which revealed the presence of both CANX and procollagen within autophagosomes (Forrester et al., 2019).

Both CANX and FAM134B have also been found to play a key role in the degradation of proteasome-resistant α1-antitrypsin Z (ATZ). ATZ degradation bypasses autophagosome sequestration and is directly targeted to the lysosome. CANX binds FAM134B and segregates ATZ polymers in ER subdomains of higher membrane curvature. FAM134B clustering induces ER membrane fragmentation into a single-membrane vesicle, encircling the misfolded ATZ cargo. The vesicle is then docked to Rab7/LAMP1-positive lysosomes through interactions that require the FAM134B LIR and LC3 on the target membrane. ATZ polymers are released into the lysosome through membrane-membrane fusion, mediated by ER-resident and lysosomal SNAREs STX17 and VAMP8, respectively. Interestingly, compared with FAM134B-driven ER-phagy, ATZ-positive vesicle formation from the ER membrane does not require the FAM134B LIR nor the early autophagy machinery, as FAM134B-positive ATZ vesicles are still formed in their absence but are unable to be delivered to the lysosome (Fregno et al., 2018). It remains to be determined how LC3 lipidation on Rab7/LAMP1-positive lysosomes is regulated and coordinated with ATZ vesicle formation.

Degradation of pro-collagen and ATZ polymers can also be achieved via the proteasome through ERAD. Whether these misfolded proteins will be targeted into the proteasome or the lysosome, will be determined by the mannose and glucose processing of N-glycans (Fregno et al., 2021).

*Akita*, a form of proinsulin in which the seventh cysteine of its A chain is mutated to threonine, causing its aggregation while also trapping the WT proinsulin (Liu et al., 2007), is also degraded through the lysosome. *Akita* is segregated into ER tubules at sites distinct to ER-exit sites, where proinsulin is usually targeted for secretory pathways. *Akita* requires RTN3L, LC3, Lunapark (LNPK), another protein involved in the maintenance of the ER morphology, and SEC24C, a subunit of the COPII complex, for its degradation. Importantly, a healthy tubular ER network is essential for the sequestration of *Akita* into autophagosomes. Conclusively, *Akita* is degraded in the lysosome through RTN3L-mediated ER-phagy of ER tubules, thereby releasing WT proinsulin for its secretion (Cunningham et al., 2019; Parashar et al., 2021).

### ER remodeling in health and disease

Preserving the integrity of the ER is essential for cellular fitness and homeostasis. With ER remodeling being central to ER-associated cellular functions, it is of little surprise to learn that various human disorders have been linked to defects in ER membrane remodeling. Furthermore, ER remodeling takes on specialized roles in different cell types, depending on the cellular need and function. In muscle cells, sarcoplasmic reticulum, a specialized type of smooth ER, allows efficient storage of cellular Ca^2+^ and maintains its concentration, thereby regulating muscle contraction (Rossi et al., 2008). In neurons, the ER predominantly comprises smooth tubules, with only some patches of rough ER. This tubular ER network is spread throughout the neuron and specifically shaped to adapt to the narrow axonal space. Its main functions include lipid and glucose metabolism and Ca^2+^ dynamics (Öztürk et al., 2020). Mutations in ER-shaping proteins in neurons can lead to a number of neurodegenerative disorders. Loss-of-function mutations in two RHD-containing ER-phagy receptors, FAM134B and ATL3, lead to hereditary sensory neuropathies (HSAN) caused by the decreased survival of sensory and autonomic neurons (Kornak et al., 2014; Kurth et al., 2009). Both of these mutations render the proteins unable to participate in membrane remodeling events, resulting in non-functional and structurally disordered ER, thus leading to progressive sensory loss as well as distal muscle weakness and wasting.

ER membrane-shaping proteins, such as RTN2, ATL1, Spastin, REEP1, and ARL6IP1, have been found to cause an inherited neurological disorder termed hereditary spastic paraplegia (HSP), a disease presented by muscle spasticity, outlining the critical nature of functional ER membrane dynamics for neuron survival (Hübner and Kurth, 2014).

Furthermore, miscommunication between the ER and other organelles is frequently a consequence of a defect in ER remodeling, and it has been linked with metabolic (diabetes, pulmonary hypertension, and cardiac pathologies [López-Crisosto et al., 2015]) and neurological (Parkinson’s disease, amyotropic lateral sclerosis [ALS], and Alzheimer’s disease [Bernard-Marissal et al., 2015, 2018; Öztürk et al., 2020]) diseases and disorders.

FAM134B and Sec62 have been found to be associated with cancer. FAM134B is thought to act as a tumor suppressor, with its mutations frequently observed in colorectal carcinoma and esophageal squamous cell carcinoma. Amplification of SEC62 is assumed to induce ER stress tolerance in tumor cells, thus likely inducing high metastatic potential in these cancers. and SEC62 if often found to be amplified in non-small cell lung cancer, prostate cancer, and others (Hübner and Dikic, 2020).

ER remodeling plays an important role in pathogen infections. Virus genome replication, assembly, morphogenesis, and egress all rely on ER membrane rearrangements (Ravindran et al., 2016). In bacteria such as *Legionella pneumophila*, ER remodeling promotes their replication. Initially, ATL3 has been found to contribute toward the formation of *Legionella*-containing vacuoles (LCV) by driving ER remodeling upon *Legionella* infection (Steiner et al., 2017). Furthermore, *Legionella pneumophila* utilizes a novel, non-canonical type of ubiquitination, termed phosphoribosyl-dependent ubiquitination (PR-Ub), to hijack host cellular processes (Bhogaraju et al., 2016; Qiu et al., 2016). The SidE family of proteins, *Legionella* pathogenic effectors that catalyze PR-Ub, target the ER-phagy receptors FAM134B, RTN3, and TEX264, as well as other ER-shaping proteins RTN4 and LNPK, and drive ER membrane remodeling for their own benefit. This results in the formation of LCVs, which promotes bacterial proliferation (Shin et al., 2020).

## Concluding remarks

Understanding the structure and function of the ER has been of interest to researchers for a number of decades. The ER plays an essential role in cellular homeostasis and is a hotspot for a variety of key cellular functions. As such, the ER has developed a number of quality control processes such as ERAD or ER-phagy to cope with stress and allow quick recovery thereafter. ER membrane remodeling is a common event in these processes, but current understanding about the mechanisms underpinning ER remodeling remains scarce. Expanding on this knowledge has gained momentum in recent years, following the discovery of RHD-containing ER-phagy receptors that not only drive ER-phagy, but have a significant effect on ER membrane dynamics and morphology. New discoveries in the field of ER remodeling will shed light on physical mechanisms underlying the ER quality control processes and provide critical insights and information about developing therapeutic and diagnostic approaches required for disorders caused by the malfunction of the ER-shaping machinery.
